# Structure-based virtual screening methods for the identification of novel phytochemical inhibitors targeting furin protease for the management of COVID-19

**DOI:** 10.3389/fcimb.2024.1391288

**Published:** 2024-06-11

**Authors:** Prashant Kumar Tiwari, Mandeep Chouhan, Richa Mishra, Saurabh Gupta, Anis Ahmad Chaudhary, Mohammed Al-Zharani, Ashraf Ahmed Qurtam, Fahd A. Nasr, Niraj Kumar Jha, Kumud Pant, Mukesh Kumar, Sanjay Kumar

**Affiliations:** ^1^ Biological and Bio-computational Lab, Department of Life Sciences, Sharda School of Basic Sciences and Research, Sharda University, Greater Noida, Uttar Pradesh, India; ^2^ Department of Computer Engineering, Parul University, Gujarat, India; ^3^ Department of Biotechnology, GLA University, Mathura, Uttar Pradesh, India; ^4^ Department of Biology, College of Science, Imam Mohammad Ibn Saud Islamic University (IMSIU), Riyadh, Saudi Arabia; ^5^ Centre for Global Health Research, Saveetha Medical College, Saveetha Institute of Medical and Technical Sciences, Saveetha University, Chennai, India; ^6^ School of Bioengineering & Biosciences, Lovely Professional University, Phagwara, India; ^7^ Department of Biotechnology, Sharda School of Engineering and Technology, Sharda University, Greater Noida, Uttar Pradesh, India; ^8^ Department of Biotechnology, Graphic Era Deemed to Be University, Dehradun, Uttarakhand, India; ^9^ Department of Biophysics, All India Institute of Medical Sciences, New Delhi, India

**Keywords:** SARS-CoV-2, furin protein, virtual screening, molecular dynamics (MD) simulation, phytochemical inhibitors

## Abstract

The coronavirus disease 2019 (COVID-19), caused by the severe acute respiratory syndrome coronavirus 2 (SARS-CoV-2) virus, is a highly contagious respiratory disease with widespread societal impact. The symptoms range from cough, fever, and pneumonia to complications affecting various organs, including the heart, kidneys, and nervous system. Despite various ongoing efforts, no effective drug has been developed to stop the spread of the virus. Although various types of medications used to treat bacterial and viral diseases have previously been employed to treat COVID-19 patients, their side effects have also been observed. The way SARS-CoV-2 infects the human body is very specific, as its spike protein plays an important role. The S subunit of virus spike protein cleaved by human proteases, such as furin protein, is an initial and important step for its internalization into a human host. Keeping this context, we attempted to inhibit the furin using phytochemicals that could produce minimal side effects. For this, we screened 408 natural phytochemicals from various plants having antiviral properties, against furin protein, and molecular docking and dynamics simulations were performed. Based on the binding score, the top three compounds (robustaflavone, withanolide, and amentoflavone) were selected for further validation. MM/GBSA energy calculations revealed that withanolide has the lowest binding energy of −57.2 kcal/mol followed by robustaflavone and amentoflavone with a binding energy of −45.2 kcal/mol and −39.68 kcal/mol, respectively. Additionally, ADME analysis showed drug-like properties for these three lead compounds. Hence, these natural compounds robustaflavone, withanolide, and amentoflavone, may have therapeutic potential for the management of SARS-CoV-2 by targeting furin.

## Introduction

Severe acute respiratory syndrome coronavirus 2 (SARS−CoV−2), a highly contagious virus causing severe respiratory illness, was first detected in China in December 2019, which later caused the outbreak of coronavirus disease 2019 (COVID-19) (Negahdaripour et al., 2022). WHO has documented 774 million confirmed cases and seven million deaths as of 21 January 2024 (COVID-19 Epidemiological Update - 29 September 2023). Coronavirus is a member of the β-coronavirus genus, which is closely related to SARS-CoV and bat coronaviruses. In comparison to SARS-CoV and MERS-CoV, it has been observed that COVID-19 spreads more rapidly among humans than both of them, and for this reason, WHO declared it an epidemic in March 2020 (Gao et al., 2020; Nagoor Meeran et al., 2020). Coronavirus represents a single-stranded positive-sense virus, characterized by a genome size ranging between 26 and 32 kilobases (kb), spherical shape Structure, and a size of 80 to 160 nanometers (nm) in length. This virus is composed of four structural proteins, namely the spike (S), envelope (E), membrane (M), and nucleocapsid (N) (Nagoor Meeran et al., 2021; Ravi et al., 2022).

The S protein of the coronavirus, a trimeric glycoprotein, plays an important role in infecting the host and consists of two distinct functional subunits known as S1 and S2. More precisely, the S1 subunit facilitates binding to the human protein angiotensin converting enzyme 2 (ACE2) receptor, while the S2 subunit plays a key role in coordinating the fusion machinery process (Millet and Whittaker, 2015; Karn et al., 2021). The functional activation of S protein occurs mainly after proteolytic cleavage by host proteases such as trypsin, cathepsin B, and cell surface-located transmembrane protease serine 2 (TMPRSS2). These proteases play an important role in cleaving the S protein, thereby facilitating viral attachment to host cells (Letko et al., 2020; Shokeen et al., 2022; Uma Reddy et al., 2022). Researchers have revealed that glycoprotein processing increases the virulence of the disease in the host. The successful inhibition of the protease enzyme can significantly inhibit virus transmission. Protease functionality is important in enabling the fusogenic ability of viruses, a critical step that facilitates the entry of enveloped viruses into host cells (Zhou et al., 2015).

The S1 subunit contains a receptor binding domain (RBD) that is responsible for specific interactions with the ACE2 receptor, facilitating viral entry. Furthermore, the S protein of the virus must undergo proteolytic activation at the junction between S1 and S2. This activation causes the dissociation of S1 and triggers significant structural changes in S2 (Shang et al., 2020). This cleavage is a prerequisite for subsequent cleavage at the S2′ site, which is important for activating the fusion machinery of the virus. Both cleavage events are necessary to initiate the membrane fusion process, a critical step in viral entry in which the viral envelope fuses with the host cell membrane, releasing genetic material (Jackson et al., 2022). In a study, it was found that within the S subunit of SARS-CoV-2, a unique site exists Arg–Arg–Ala–Arg (R-R-A-R), which is cleaved by furin protease (Coutard et al., 2020), that exposes RBD to bind host ACE2 receptor (Essalmani et al., 2022), leading to viral entry into the host cells. Hence, targeting the furin protease could be a potential strategy to reduce SARS-CoV-2 infectivity and enhance immune responses.

A variety of drugs have been used to treat COVID-19, mainly antimalarial, antiviral drugs, some immunosuppressants, and convalescent plasma therapy. However, a number of side effects have been reported with these especially severely affected COVID-19 people, which is a matter of concern. For example, individuals with serious medical conditions undergoing remdesivir treatment have faced serious adverse effects, such as kidney dysfunction and low blood pressure (Wang et al., 2020). In various independent studies, evidence indicates that standard medications may cause interactions either between drugs or between nutritional components and drugs in individuals severely affected by COVID-19. These interactions produce potential adverse effects for patients (Ağagündüz et al., 2021). As a result, it becomes imperative to adopt safer alternative strategies using plant-derived compounds. Several scientific studies have acknowledged the effectiveness of plant-based compounds and their secondary metabolites in combating SARS-CoV. Some *in silico* studies have indicated that small compounds derived from plants may also be effective against SARS-CoV-2. Additionally, *in vitro*, cell culture, and *in vivo* clinical studies have further strengthened the potential of these compounds in suppressing COVID-19 (Uma Reddy et al., 2022). Natural products could serve as an important source of new medicines that help to prevent and manage symptoms of COVID-19. Various compounds, such as flavonoids, polyphenols, alkaloids, and polysaccharides derived from plants and mushrooms, which have been traditionally used in the treatment of infectious diseases, are currently being used in the management of COVID-19. This is attributed to their immune-boosting, antimicrobial, and anti-inflammatory properties. These phytochemical compounds exhibit the ability to inhibit virus entry and resistance, as well as contribute to the regulation of the immune-inflammatory response against COVID-19 (Isidoro et al., 2022).

A previous study has reported that compounds such as d-Arg-based peptides and α1-antitrypsin portland inhibit furin, although the peptides face degradation and absorption issues, leading to increased stability (Devi et al., 2022). Synthetic macrocyclic peptidomimetics address these challenges, aiming to improve stability and effectively inhibit furin by binding to its active site (Devi et al., 2022). In a study conducted by Cheng et al. (2020), furin inhibitors such as CMK and naphthofluorescein effectively inhibit cleavage and syncytium formation in SARS-CoV-2-infected cells by reducing virus production and associated cytopathic effects and showing promising antiviral effects. Additionally, CMK not only inhibits virus entry similar to chemostat but also inhibits spike cleavage and syncytium formation, whereas naphthofluorescein primarily inhibits viral RNA transcription. These findings highlight the considerable potential of furin inhibitors as effective antiviral agents for both the prevention and treatment of SARS-CoV-2 infection (Cheng et al., 2020). Here, utilizing an *in silico* approach, we have proposed plant-derived natural compounds as potential inhibitors of furin protease that could be a promising candidate to block the viral entry into human cells.

## Methods

### Protein optimization

The native crystal structure of the human furin protein PDB ID-5MIM with a good resolution of 1.90 Å was obtained from the RCSB Protein Data Bank (Shokeen et al., 2022) (https://www.rcsb.org/). For structure-based virtual screening, initially, the protein was processed in several steps, such as eliminating crystallized water molecules and heteroatoms, as well as assigning Gasteiger charges and adding polar hydrogen atoms. To compare the studies of the selected candidates, the naphthofluorescein inhibitor was used as a reference compound (Cheng et al., 2020).

### Preparation of natural compound library

Based on their antiviral properties, 408 natural phytochemicals were selected from the literature review (Singh et al., 2023). The three-dimensional (3D) model structures of these phytochemical compounds in SDF format were obtained from the PubChem database (https://pubchem.ncbi.nlm.nih.gov/). These 3D SDF phytochemical data were combined to create a library using Progenesis SDF Studio (Progenesis SDF Studio, n.d.).

### Receptor grid generation and high-throughput virtual screening using the PyRx program

Molecular screening was conducted using PyRx software, employing the AutoDock Vina wizard as the docking engine. The library of phytochemical compounds underwent energy minimization using the UFF force field, followed by screening against the furin protein via PyRx.

A grid was specifically generated for the amino acid residues interacting with the S protein of the virus, as identified in the literature. These residues include Arg268, Glu271, Glu272, Leu269, Asp233, Th232, Ala234, Gly265, Phe275, Glu236, Asp264, Tyr308, Ala267, Asn243, Arg276, Asn387, Glu230, Gln280, His248, Lys449, His145, Lys386, Gly146, Asn245, Asp168, His246, Val148, and Arg220 (Shokeen et al., 2022), thus forming the basis for the grid generation. The grid parameters were set as follows: center *x* = 32.65, *y* = −26.52, *z* = 2.63 and size *x* = 56.36, *y* = 31.64, *z* = 44.81. Throughout this process, the ligands were considered flexible. The ligand exhibiting the most negative binding energy score was considered to have the highest binding energy. On the basis of the binding energy, the best 10 compounds were chosen for redocking to evaluate our best candidates against furin protease as compared to the reference molecule.

### Redocking of top hit molecules and calculation of inhibition constant

The top 10 compounds obtained from the PyRx result, as well as the reference molecule, were redocked for validation using AutoDock Tool 4.2. Utilizing Lamarckian genetic algorithm, ligands optimal poses at the furin protein binding site were determined (Morris et al., 2009). Default settings were followed to find the best docking confirmation. Binding energies and inhibition constants (Ki) against human furin were extracted and sorted. Consistent pattern in docking data for the top small molecules was emerged, and each ligand generated fifty conformations with predicted Ki values. Anticipated binding was computed using AutoDock, ensuring robust computational techniques (Morris et al., 2009). This methodological approach provided a comprehensive assessment of ligand binding potential and facilitated further analysis for drug discovery efforts targeting furin protease.

### Molecular dynamics simulation

We performed molecular dynamics (MD) simulations on the protein–ligand docked complex using the Desmond module of Schrödinger Maestro (Price and Brooks, 2004; Bowers et al., 2006) for a duration of 100 nanoseconds (ns). The aim was to analyze the stability of the protein–ligand complex in a dynamics system. A cubic box with dimensions of 10 Å × 10 Å × 10 Å was employed to incorporate these complexes, utilizing the transferable intermolecular potential three-point solvent model (TIP3P) (Price and Brooks, 2004). To replicate the physiological conditions, the simulation system was balanced by introducing counter ions (Na^+^ or Cl^−^) into a 0.15-mol/L salt solution. We employed the steepest descent and conjugate gradient methods to reduce the energy of the pre-prepared system. The system was initially reduced in size by applying constraints on the solute to optimize interaction, up to a maximum of 2,000. We used a convergence criterion of 1.0 kcal/mol/Å. The Broyden–Fletcher Goldfarb–Shanow (LBFGS) method was employed to complete the energy reduction step after constructing the system. The algorithms utilized the most efficient descent steps and were distinguished by their restricted memory capacity (Kaczor et al., 2015). In addition, the Berendsen NVT and NPT ensembles were used to thermally relax the minimized complexes at a temperature of 300 K. The relaxation time for the NVT ensemble was 1 ps, while the relaxation time for the NPT ensemble was 2 ps at a pressure of 1.013 bars atm. We maintained the isothermal ensemble (NPT) using this relaxation technique. The constant pressure and temperature were maintained through the use of the Nose–Hoover thermostat and the Martyne–Tobias–Klein barostat techniques, which were employed to model the entire system. Reversible reference system propagator algorithm (RESPA) integrator was used throughout the simulations. In the most recent simulation run, we calculated the bonding interactions for a time interval of 2 femtoseconds. We used the particle mesh Ewald (PME) technique to calculate the extensive electrostatic interactions throughout the simulation. We conducted the entire procedure of data calculation with the OPLS3 force field for all atoms. The molecular dynamics simulation trajectories were utilized to quantify the root mean square deviation (RMSD), root mean square fluctuation (RMSF), and protein–ligand interaction profiling for all potential docked ligand complexes with the target protein (Harder et al., 2016). Maestro v12.6 tool in Schrodinger Suite 2020–4’s free academic version used for the generation of protein–ligand interaction profiling.

### Free binding energy calculations by Prime MM/GBSA

We computed the binding free energy of the top hit complexes for furin proteins using Schrödinger Prime and VSGB 2.0. We estimated the Δ*G*-binding energy of each protein–ligand complex by sampling 100 frames from the final 10 ns of the simulation. Numerous parameters, including hydrogen bonding, Coulombic, lipophilic, and van der Waals contacts, as well as generalized-born electrostatic solvation, were computed as components for the MM/GBSA analysis (Gupta et al., 2022; Kumar et al., 2023). We ascertained the standard deviation upon estimating the mean energy value of MM/GBSA. We then computed the net binding free energy using the thermal mmgbsa.py tool.


$${\text{MM}/\text{GBSAΔG}}_{\text{bind}}={\text{ΔG}}_{\text{complex}}-{\text{ΔG}}_{\text{protien}}-{\text{ΔG}}_{\text{ligand}}$$

The symbols Δ*G* complex, Δ*G* protein, and Δ*G* ligand represent the free energies of the hit ligand–protein complex, protein, and ligand, respectively. A decreased binding free energy signifies an enhanced binding affinity of the small molecule for the protein–ligand combination.

### Drug-likeness and ADME properties

The pharmacokinetic properties of the natural plant compounds obtained from the docking results were evaluated considering ADME peculiarities. These peculiarities indicate the extent to which compounds can potentially serve as primary drugs. The drug-like properties of the compounds were evaluated using the SwissADME online tool (http://www.swissadme.ch). This tool evaluates various physical properties, including physicochemical properties, water solubility, drug similarity, kinetics, medicinal chemistry, lipophilicity, etc. This evaluation helps to determine whether these compounds follow Lipinski’s rule of five (molecular weight < 500, QPlogPo/w < 5, DonorHB < 5, acptHB < 10) (Chen et al., 2020). The purpose of choosing these characteristics was to investigate how they assess their effect on cell membrane permeability (pCaco-2 and pMDCK), qualitative oral absorption (% human oral absorption), as well as their impact on the distribution, metabolism, and toxicity of the compounds.

## Results and discussion

### Docking studies

A library of 408 natural compounds, previously identified in the literature for their antiviral properties, was utilized for further investigation. These compounds were screened utilizing high-throughput virtual screening against a furin protease that interacts primarily with the S subunit of the SARS-CoV-2 spike protein (**Supplementary Table S1**). Initially, we extracted the furin protease inhibitor from its crystal complex (Douglas et al., 2022) and docked it into a defined binding pocket for screening using default parameters. The docked pose of the inhibitor was superimposed on the original crystal conformation to validate the docking protocol (**Supplementary Figure S1**). The RMSD was less than 2 Å. Three compounds outperformed the reference molecules in terms of docking scores against furin protease, making them top hits for further *in silico* validation. Each compound in the library exhibited promising binding energies (−10.8 kcal/mol to −2.5 kcal/mol). The top three, robustaflavone (−10.7 kcal/mol), amentoflavone (−10.5 kcal/mol), and withanolide (−10.4 kcal/mol), demonstrated strong binding. They were validated by redocking, confirming their affinity, with scores of −10.6 kcal/mol, −10.5 kcal/mol, and −10.4 kcal/mol, respectively. The reference compound scored −10.2 kcal/mol.

### Docking analysis of robustaflavone

**Figure 1:**
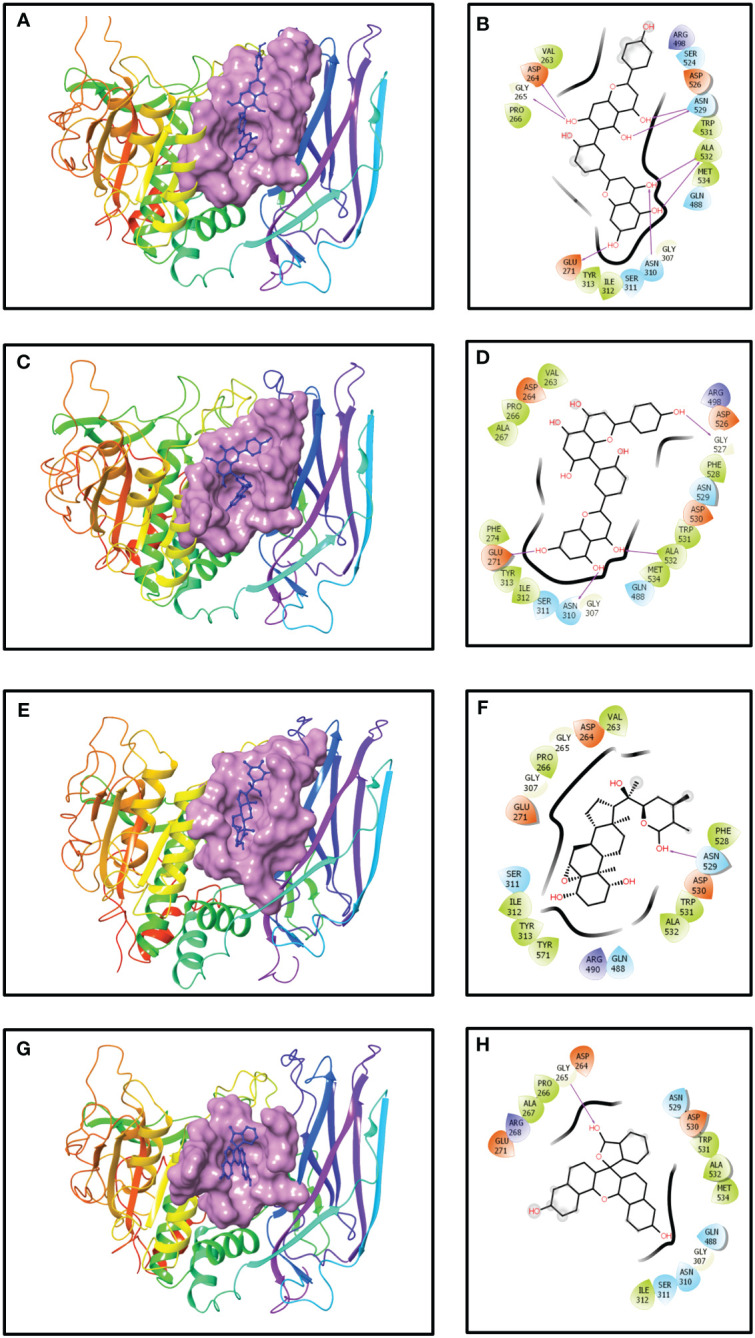
Three- and two-dimensional docked complexes of the selected phytochemical-derived natural compounds, i.e., **(A, B)** robustaflavone, **(C, D)** amentoflavone, **(E, F)** withanolide, and **(G, H)** reference complexes, i.e., naphthofluorescein, showing binding on the active site of the furin protein. Whereas in two-dimensional structures, H-bond formation (pink arrows), hydrophobic interaction (green), polar residue (blue), negative residue (red), glycine (grey), and salt bridge (red and blue) interactions are logged for docked complexes of furin protein with selected phytochemical compounds.

The outcome of the molecular docking analysis of robustaflavone demonstrated a notable docking score of −10.6 kcal/mol towards the binding pocket, exhibiting significant and strong binding interaction with the human furin protein (**Table 1**). The protein residues bound to the ligand showed the polar interaction with Asn529 and Asn310, the hydrophobic interaction with Ala532, and the negative residual interaction with Asp264 and Glu271 were involved in the formation of hydrogen bonds. Additional hydrogen bonds were also observed with the hydroxyl group of the ligand and Gly265 of the furin. Polar contacts were also identified, such as Ser311, Asn310, Gln488, Ser524, and Asn529. Additionally, hydrophobic interactions involving Val263, Pro266, Tyr313, Ile312, Trp531, Ala532, and Met534 were observed. Furthermore, ionic interactions were detected with Asp264, Glu271, Arg498, and Asp526 residues (**Figures 1A, B**). The reference compound and robustaflavone compound shared almost all amino acids, such as the following: Asp264, Gly265, Pro266, Glu271, Ile312, Ser311, Asn310, Gly307, Trp531, Ala532, Met534, and Asn529.

**Table 1:** Binding affinity and redocking score calculated by chimera and estimated inhibition constant (KI) energy of screened compounds.

S. No.	Compound CID	Ligand	Binding affinity (kcal/mol)	Redocking score	Interacting residues in docking	Estimated inhibition constant (Ki)
1.	5281694	Robustaflavone	−10.7	−10.6	Val263, Asp264, Gly265, Pro266, Glu271, Tyr313, Ile312, Ser311, Asn310, Gly307, Arg498, Ser524, Asp526, Asn529, Trp531, Ala532 Met534, Gln488	96.20 picomolar (pM)
2.	5281600	Amentoflavone	−10.5	−10.5	Val263, Asp264, Pro266,Ala267,Phe274, Glu271, Tyr313, Ile312,Ser311, Asn310, Gly307, Arg498, Asp526, Gly527, Phe528, Asn529, Asp530, Trp531, Ala532, Met534,and Gln488	514.69 pM
3.	53477765	Withanolide	−10.4	−10.4	Val263, Asp264, Gly265, Pro266, Gly307, Glu271, Ser311, Ile312, Tyr313, Tyr571,Gln488, Arg490, Phe528, Asn529, Asp530, Trp531,and Ala532	539.40 pM
4.	3124834	Naphthofluorescein (reference compound)		−10.2	Asp264, Gly265, Pro266, Ala267, Arg268, Glu271, Ile312,Ser311, Asn310, Gly307, Glu488, Asn529, Asp530, Trp531, Ala532,and Met534	30.89 nanomolar (nM)

### Docking analysis of amentoflavone

The results from the molecular docking analysis revealed a considerable docking score of the compound amentoflavone for the binding pocket, indicating substantial and robust binding interactions with the human furin protein. This interaction was quantified by a docking score of −10.5 kcal/mol (**Table 1**). The benzoate-hydroxyl group of the compound amentoflavone displayed hydrogen bonds with the main chain residues Glu271, Asn310, Gly527, and Ala532. Additionally, hydrophobic residues Val263, Pro266, Ala267, Phe274, Tyr313, Ile312, Phe528, Trp531, Ala532, and Met534 were identified. The compound amentoflavone exhibited polar interactions with various amino acids, such as Ser311, Asn310, Gln488, and Asn529, and also exhibited ionic interactions with Asp264, Glu271, Arg498, Asp526, and Asp530, which were surrounded by phenyl residues (**Figures 1C, D**). In both amentoflavone and reference compounds, almost all residues such as Asp264, Pro266, Ala267, Glu271, Ile312, Ser311, Asn310, Gly307, Asn529, Asp530, Trp531, Ala532, and Met534 were present.

### Docking analysis of withanolide

Molecular docking analysis revealed an impressive binding affinity, determined by a docking score of −10.4 kcal/mol (**Table 1**). Furthermore, the resulting complex of withanolide and furin displayed polar interactions with the amino acid residues, such as Ser311, Gln488, and Asn529. In addition to these, the complex was supported by hydrophobic interactions within the protein structure, particularly with Val263, Pro266, Ile312, Tyr313, Phe528, Trp531, Ala532, and Tyr571. Furthermore, the ionic interaction was displayed by some specific amino acid residues, including Asp264, Glu271, Arg490, and Asp530 (**Figures 1E**, **F**). Among these, most amino acid residues, such as Asp264, Gly265, Pro266, Glu271, Ser311, Ile312, Asn529, Asp530, Trp531, and Ala532, were also present in both reference compound and withanolide.

### Docking analysis of naphthofluorescein (reference compound)

It was demonstrated that the naphthofluorescein compound, acting as a reference molecule, established a single hydrogen bond with Gly265 of the furin protein during the binding process. In addition, several other contacts were stabilized, including polar amino acids like Asn310, Ser311, Gln488, and Asn529, as well as hydrophobic amino acids such as Pro266, Ala267, Ile312, Trp531, Ala532, and Met534. Additionally, the furin protein displayed ionic interactions with Asp264, Arg268, Glu271, and Asp530 (**Figures 1G, H**). The docking score was found to be −10.2 kcal/mol (**Table 1**).

### ADME analysis

The inherent properties of phytochemical compounds used in medical applications are of utmost importance. These include characteristics such as drug-likeness and pharmacological properties like solubility, permeability, and metabolic stability. Therefore, the absorption, distribution, metabolism, and excretion (ADME) properties of the top three selected compounds, robustaflavone, amentoflavone, and withanolide, were predicted using SwissADME. ADME was used to predict the biochemical properties of these compounds (**Supplementary Table S2**). Cytochrome P450 2D6 (CYP2D6) enzymes show an essential role in the metabolism of drugs and xenobiotics. Inhibition of CYP2D6 may alter interactions between various drugs. An interesting fact is that the top three compounds selected were not found to inhibit CYP2D6, despite their effects on other cytochromes, according to SwissADME. Following Lipinski’s rule of five, two compounds, namely robustaflavone and amentoflavone, show two violations each, while the compound withanolide shows zero violations, as mentioned in **Table 2**; **Supplementary Table S2**. Additionally, considering other rules of drug-likeness, such as Ghosh, Veber, Egan, and Muegge violations, the compound withanolide appears as an ideal candidate.

**Table 2:** ADME analysis.

Compound name	Molecular weight (g/mol)	Rotatable bonds	H-B-D	H-B-A	GI absorption	BBB permeant	log *K*_p_
Robustaflavone	538.46	3	6	10	Low	No	−6.01 cm/s
Amentoflavone	538.46	3	6	10	Low	No	−6.01 cm/s
Withanolide	470.60	2	2	6	High	No	−6.96 cm/s
Naphthofluorescein (reference compound)	432.42	0	2	5	High	No	4.73 cm/s

BBB permeant, blood–brain barrier; H-B-D, H-bond donors; H-B-A, H-bond acceptors; log P (O/W), for octanol/water (−2.0/6.5); log K_p_, skin permeation.

It is important to note that the potential drug-likeness rule does not apply to natural bioactive molecules because these molecules are recognized by the active transport system of human cells. In conclusion, the three compounds investigated may have the potential to be developed as drugs in future studies (**Table 2**; **Supplementary Table S2**).

### Molecular dynamics simulation data analysis

**Figure 2:**
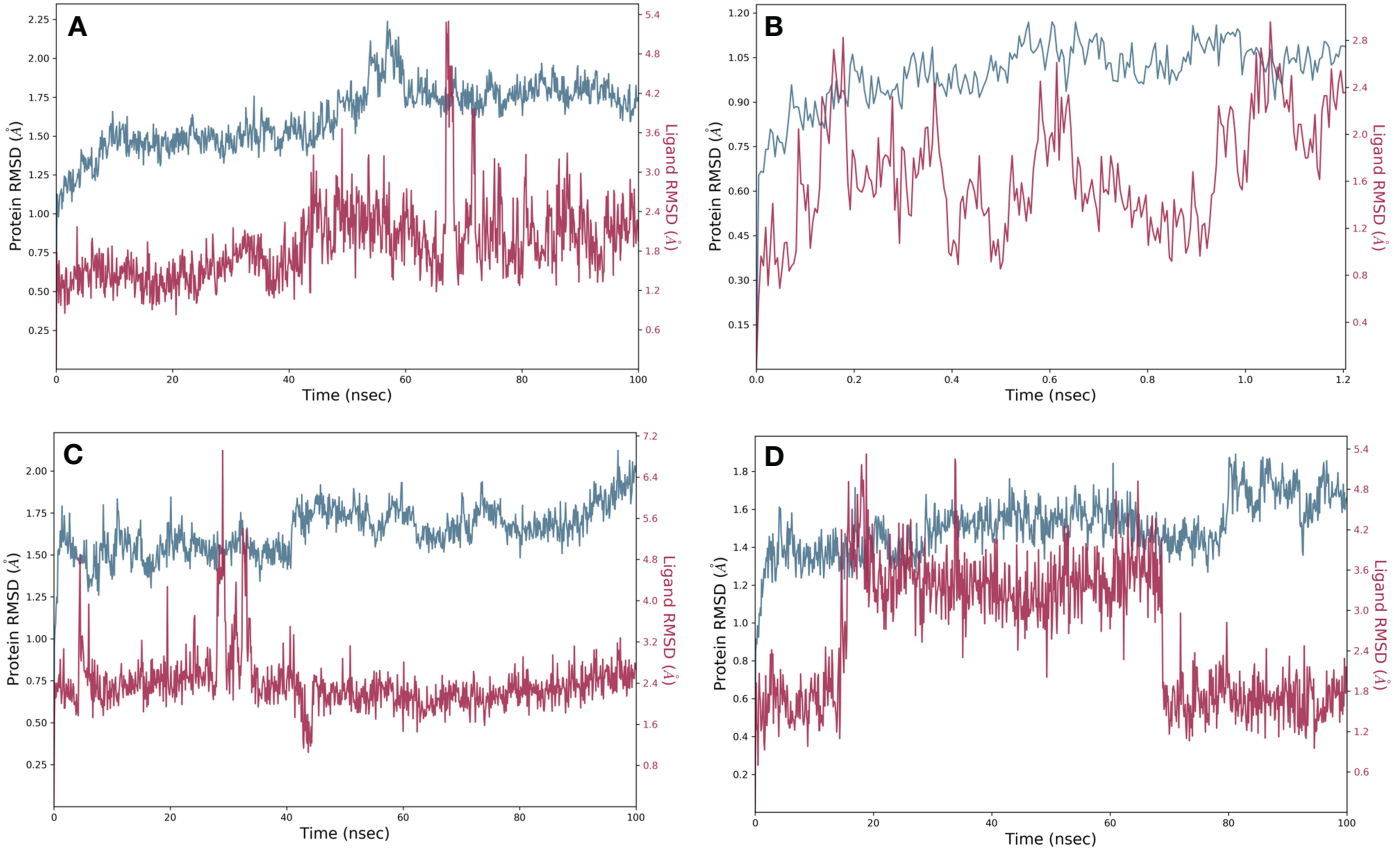
RMSD plots for the backbone atoms of furin protein and selected phytochemically derived natural compounds, i.e., **(A)** robustaflavone, **(B)** amentoflavone, **(C)** withanolide, and **(D)** reference complexes, i.e., naphthofluorescein, fit on selected target proteins were extracted from 100 ns MD simulation trajectories of different docked complexes.

We studied the dynamic perturbations within the complex and the interactions with the ligands using MD simulations. With the use of MD simulation experiments with a period of 100 ns, we aimed to evaluate the dynamic behavior and strength of the top-docked complex with the established inhibitor naphthofluorescein in the proposed catalytic site of human furin. We evaluated the stability of the complex structure by utilizing RMSD and RMSF. The simulation interaction diagram module was utilized for this specific objective. RMSD measures how stable and adaptable the structure of furin was throughout MD simulations. The data revealed reduced deviation in docked complexes that preserve steady connections in the protein’s active region. The RMSD of robustaflavone, amentoflavone, withanolide, and the reference compound (naphthofluorescein) was analyzed by comparing the positions of their alpha carbon (Cα) atoms during a duration of 100 ns. Furthermore, the RMSD plot of protein in selected complexes displayed a deviation of < 2.7 Å. Compound robustaflavone in its complex showed consistent RMSD of < 3.5 Å throughout the whole simulation, indicating its stability throughout the simulation period, but it also exhibited deviation up to 5 Å at 70 ns (**Figure 2A**). Additionally, the RMSD of compound amentoflavone in the docked complex showed stability throughout the simulation (3 Å); this compound was found to be very steady as compared to other docked complexes (**Figure 2B**). Moreover, withanolide exhibited steady behavior with reduced deviation during the simulation time (< 5 Å) and showed a deviation of 7.2 Å at 30 ns time (**Figure 2C**). Whereas, the reference compound showed a deviation of < 5.4 Å, exhibiting significant divergence at around 20 ns as well as approximately 70 ns (**Figure 2D**). In comparison to the reference compound, our selected compound exhibits better RMSD.

**Figure 3:**
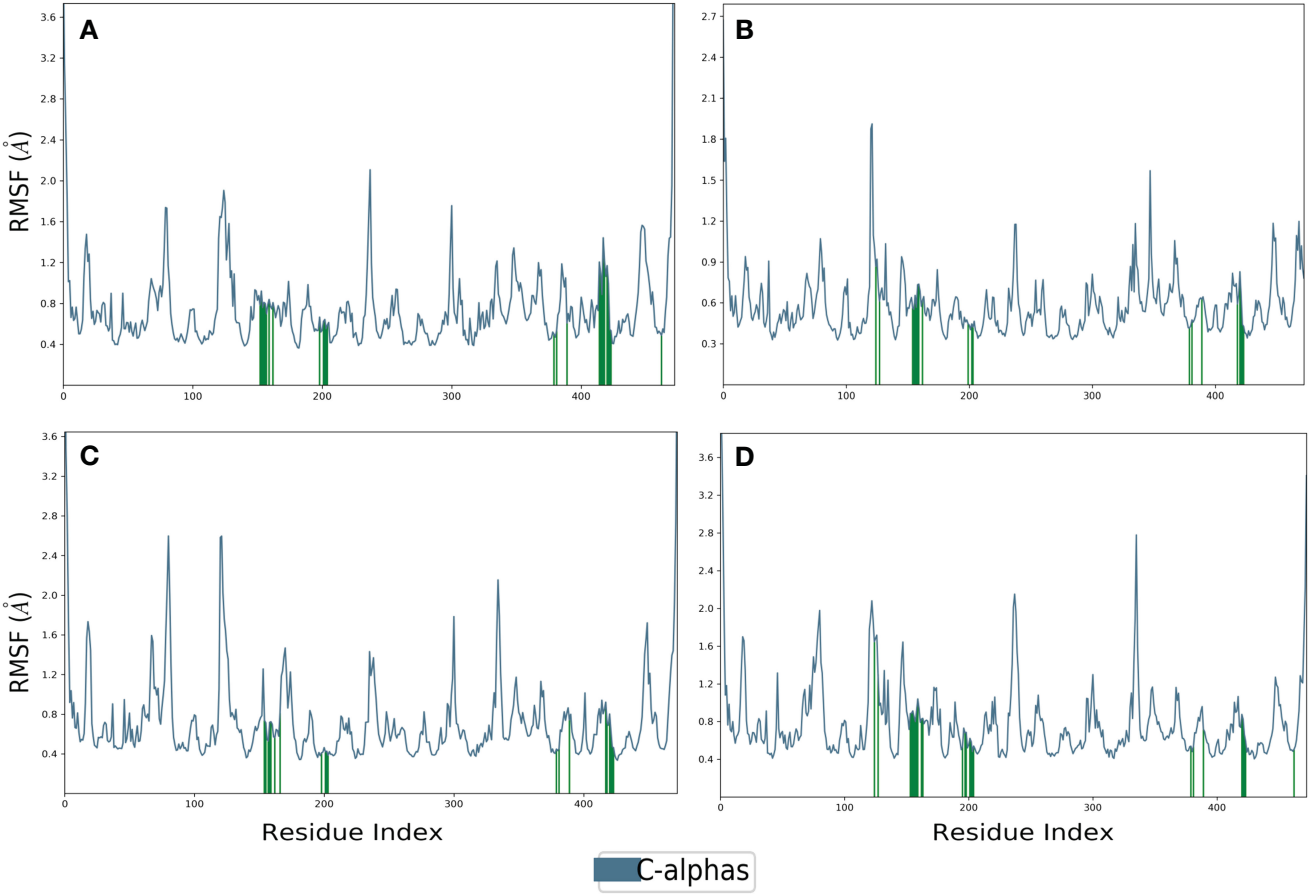
RMSF plot generated for the furin protein docked with selected phytochemical compounds, i.e., **(A)** robustaflavone, **(B)** amentoflavone, **(C)** withanolide, and **(D)** reference complexes, i.e., naphthofluorescein.

**Figure 4:**
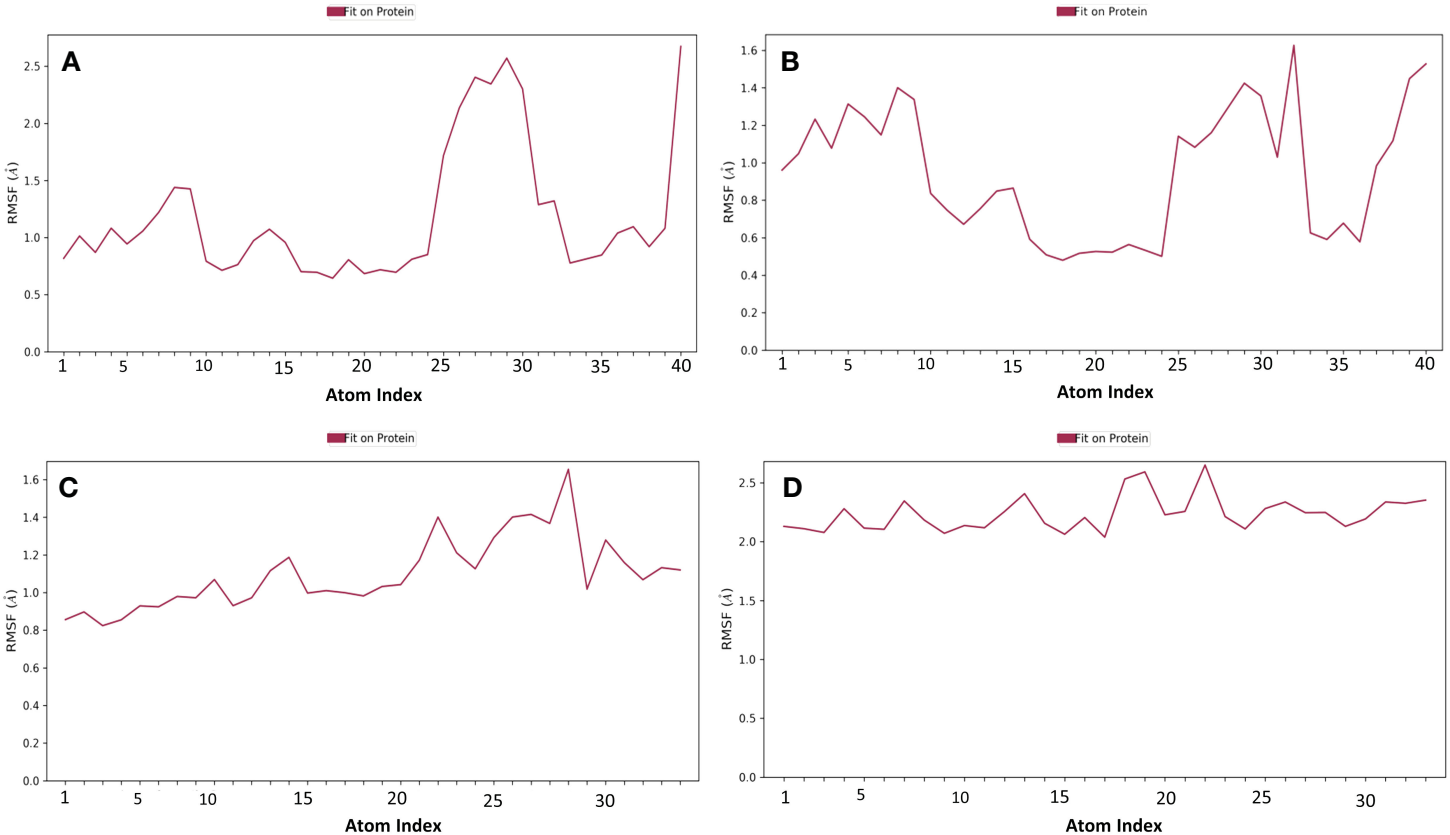
RMSF plot generated for the docked phytochemical compounds, i.e., **(A)** robustaflavone, **(B)** amentoflavone, **(C)** withanolide, and **(D)** reference complexes, i.e., naphthofluorescein, fit in the furin protein during 100 ns molecular dynamics simulation interval.

Proteins typically display an intrinsically dynamic nature, characterized by motion in their side chains and alterations in their three-dimensional structure. The structural differences have a significant impact on conformational variations, as seen by RMSF. The plots were utilized to examine the specific structural alterations in the Cα atoms of furin protein and evaluate the impact on the binding of ligands (**Figure 3**). The protein in all the complexes exhibited much reduced fluctuations, except in the terminal regions. Also, the chosen compounds had acceptable changes in their atoms (**Figure 4**). On the other hand, the protein in the reference complex remained the same, and the atoms of the reference compound in the docked complex changed by less than 3 Å (**Figures 3D**, **4D**). The last snapshot of MD simulations was re-evaluated to analyze any alterations in the interaction behavior of the docked complex. The findings indicated that certain interactions with the docked complex exhibited similarities, albeit with less conformational diversity and structural compactness. In order to study how the protein binds to ligands, different types of interacting forces were looked at during the simulation period. These included hydrogen bonds, polar contacts, and hydrophobic interactions. This analysis aimed to comprehend the interface region, where the ligand and the protein come into contact.

**Figure 5:**
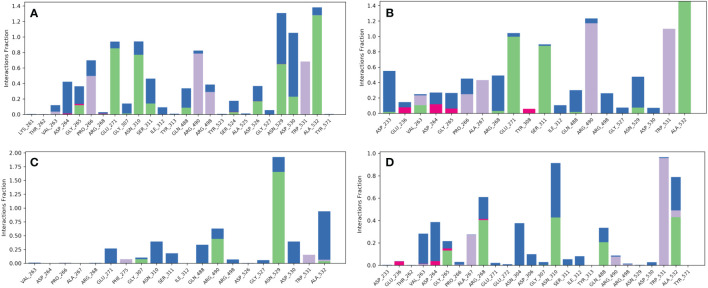
Protein–ligand interaction mapping for furin protein docked with selected phytochemical compounds, i.e., **(A)** robustaflavone, **(B)** amentoflavone, **(C)** withanolide, and **(D)** reference complexes, i.e., naphthofluorescein, extracted from 100 ns molecular dynamic simulations.

Compound robustaflavone, showed more than 70% hydrogen bonding with residues Glu271, Asn310, Asn529, and Ala532, and hydrophobic occupancy between 50% and 80% with the residues Pro266, Arg490, Arg498, and Trp531 (**Figure 5A**). Additionally, compound amentoflavone exhibited more than 90% hydrogen bonding with Glu271, Ser311, and Ala532 residues, hydrophobic occupancy of 40% with Ala267, and more than 100% with Arg490 and Trp531 (**Figure 5B**). Moreover, the compound withanolide showed hydrogen bonding with residues Arg490 and Asn529 with 50% and more than 100% of simulation time, respectively (**Figure 5C**). Whereas, the reference compound represented hydrogen bonding between 40% and 50% with residues Arg268, Asn310, and Ala532, and hydrophobic occupancy of 30% and 95% with residues Ala267 and Trp531 (**Figure 5D**). These pieces of evidence suggest that robustaflavone and amentoflavone exhibit stronger binding affinity than withanolide.

### Secondary structure dynamics

The secondary structural elements of proteins can synchronize the protein’s stiffness, which allows proteins to maintain their three-dimensional (3D) structures. The constituents of protein secondary structures preserve their three-dimensional geometry and synchronize their flexibility. Our goal was to examine how ligands affected furin in MD simulations by analyzing the secondary structure components’ dynamics. For over 100 ns, the secondary structure components showed consistent patterns in the predicted trajectory. Throughout the simulation, the furin secondary structure remained unchanged both prior to and following the binding of each ligand to furin. **Table 3** presents the analytical summary of the average number of residues involved in developing the stability of the furin secondary structure. The secondary structural changes in all the compounds caused a small drop in the number of α-helices and β-sheets during the simulation. These changes were strongly associated with the findings of RMSD and RMSF analyses, indicating a more condensed and stable docked complex.

**Table 3:** The percentage of amino acid residues that participated in the secondary structure of human furin and its complexes was calculated after 100 ns of MD trajectories.

Complex	α-Helix	β-Sheet	Total protein secondary structure elements (SSE)
**Furin-5281694**	13.49	24.62	38.11
**Furin-5281600**	14.22	23.76	37.98
**Furin-5347765**	13.10	24.17	37.26
**Furin-reference**	13.13	24.51	37.65

### Postdynamics binding free energy calculation

The process of developing a novel drug includes a comprehensive study of the molecular interaction between the potential drug and its target. A molecular structure analysis alone is insufficient for this purpose. To comprehend the multitude of elements that impact the protein–ligand binding process, researchers employ diverse methodologies. Thus, we employed thermodynamic measurements utilizing the primary MM/GBSA technique to evaluate the free binding energy of the optimized free receptors, free ligands, and ligand–receptor complexes. The MM/GBSA free energy quantifies the strength of interactions between proteins and ligands using a numerical representation. We computed the free energies of binding for the substances robustaflavone, amentoflavone, and withanolide, as well as the reference compound, using the MM/GBSA method. This study utilized energy-stabilized conformations with a simulated timescale of the last 10 ns. The energy of each of the three hit compounds and the reference compound was individually analyzed and recorded in **Table 4**. MM/GBSA data showed that the binding energy of withanolide was nearly the same as the positive control, which had a stable binding energy of −57.65 kcal/mol. The withanolide compound exhibited the greatest stability (Δ*G* = −57.2 kcal/mol) compared to all the other compounds examined, with robustaflavone (Δ*G* = −45.2 kcal/mol) coming in second, as inferred from this study.

**Table 4:** Computed MM/GBSA-binding free energies for the top HITS identified.

Complex	Binding energy	Coulomb energy	Covalent binding energy	Van der Waals energy	Lipophilic energy	DG_Bind_Solv_GB
Amentoflavone	−39.68	−10.56	1.54	−41.34	−13.72	24.53
Robustaflavone	−45.2	−11.09	1.98	−40.73	−15.81	24.85
Withanolide	−57.2	−15.04	2.85	−50.18	−16.06	28.48
Positive control, naphthofluorescein	−57.65	−25.22	2.85	−36.09	−19.41	25.64

All energy values are reported in kilocalories per mole.

## Discussion

The COVID-19 pandemic has forced scientists around the world to engage in rapid research expeditions for antiviral/vaccine development against SARS-CoV-2 for human safety. Since it takes a lot of time to develop a medicine or vaccine, it became quite challenging to find an immediate cure for the pandemic. Therefore, the fastest and most effective approach could be a repurposing option, where already approved antiviral drugs were tested against SARS-CoV-2. To understand such problems, attempts were made to predict potential antiviral drugs/compounds/natural inhibitors that can inhibit the process of SARS-CoV-2 infection at various levels (Tazeen et al., 2021).

**Figure 6:**
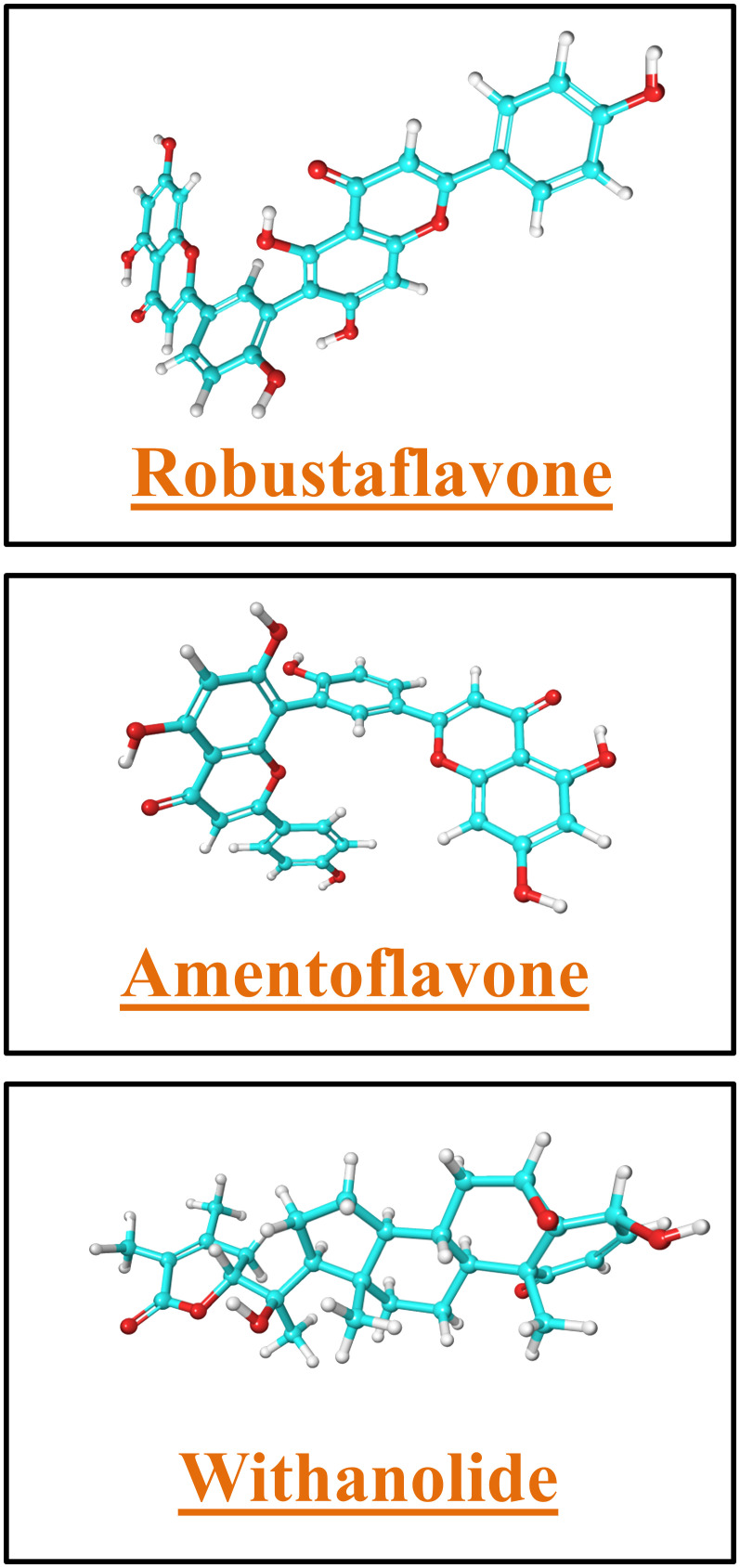
The structure of lead molecules robustaflavone, amentoflavone, and withanolide.

In this study, the focus was to analyze the structural analysis of furin, specifically its interaction with the S subunit of the spike protein. We utilized the crystal structure of human furin to identify potential compounds that could impede the interaction. The interacting amino acid residues of furin with the S protein of the virus were chosen as potential binding sites for the docking study. It is possible that furin inhibitors based on active site residues could be the most promising candidates for drug development. All of the compounds that exhibited higher docking scores were considered for further analysis. The proteins in the chosen complexes showed a variation of less than 2.7 Å in the RMSD plot. All of the selected compounds showed better RMSD when compared to the reference compound. Moreover, all of the compounds exhibited acceptable RMSF. Conversely, the protein in the reference complex remained stable, and the atoms of the reference compound in the docked complex exhibited fluctuations of less than 3 Å as well. The molecular dynamics and combinatorial docking method indicate that all three lead compounds (withanolide, amentoflavone, and robustaflavone) may disrupt furin function. **Figure 6** shows the structures of these compounds.

Robustaflavone has been shown to be a potential molecule for combating SARS-CoV-2. Beyond its role in inhibiting the furin protease, it also demonstrates potential inhibitory activities against various other targets of the virus, such as the main protease (Mpro) enzyme, which is crucial for viral replication (C et al., 2020). Moreover, robustaflavone shows promise in inhibiting the spike protein of SARS-CoV-2, a key component for viral entry into host cells (Mondal et al., 2021). Additionally, it has been explored for its potential to impede the activities of several other viral enzymes, such as papain-like protease (nsp3), 3-chymotrypsin-like protease (nsp5), RNA-dependent RNA polymerase (nsp12), helicase (nsp13), SAM-dependent 2′-*O*-methyltransferase (nsp16) along with its cofactor (nsp10), and endoribonuclease (nsp15). These enzymes play pivotal roles in various stages of the viral life cycle, including replication and transcription (de Leon et al., 2021). The ability of robustaflavone to inhibit multiple targets underscores its potential as a therapeutic agent against SARS-CoV-2.

Similarly, a study has demonstrated that amentoflavone exhibits potential efficacy in inhibiting several nonstructural proteins (NSPs) of the virus, such as Nsp1, Nsp3, Nsp5, Nsp12, and Nsp15 (Portilla-Martínez et al., 2022). In another study conducted by Lopes et al. (2022), the plant compound amentoflavone was examined for its ability to inhibit five key targets of SARS-CoV-2, including main protease (Mpro or 3CLPro), RNA-dependent protease, NSP15 endoribonuclease, S protein, and ACE2 receptor. Choudhary et al. (2023) found that withanolide A exhibits dual action, including as an antiviral agent that targets viral proteases like Mpro and PLpro and as an anti-inflammatory agent that regulates cytokine levels as well as enhancing the type-I interferon response. That evidenced that withanolide plays a crucial role in the management of SARS-CoV-2 infection (Choudhary et al., 2023).

Withanolide, robustaflavone, and amentoflavone are potential inhibitors compared to other studies. In a previous study, phytochemical compounds were used to target furin protein, and the selected compounds (limonin, eribulin, pedunculagin, genin, limonin glycoside, and betunilic acid) showed binding energies ranging from −8.7 to −7.2. kcal/mol (Vardhan and Sahoo, 2022). Additionally, Dankwa et al. (2022) employed *in silico* techniques to inhibit the furin protein using African-origin natural compounds to treat COVID-19. In their findings, they identified seven potential compounds, including quercitrin, teucrol, malvidin-3-arabinoside, *N*-*E*-caffeoyl tyramine, ZINC000085967772, pinobanksin_3-(*E*)-caffeate, and abyssinone IV, with binding scores ranging from −8.0 to −9.3 kcal/mol. Moreover, Paul et al. (2020) used Tulsi (a medicinal plant) extract in their study to inhibit SARS-CoV-2 by targeting the furin protein. Oleanolic acid obtained from this extract displayed a binding energy of −8.1 kcal/mol (Paul et al., 2020). Similarly, nefamostat is a synthetic serine protease inhibitor. It is used primarily in Japan and South Korea to treat pancreatitis as an anticoagulant during hemodialysis. Uppal et al. (2022) used a nafamostat compound to inhibit the furin protein, where they found that it displayed a binding energy of −9.1 kcal/mol. In contrast to these studies, our selected compounds showed better docking scores ranging between −10.7 and −10.4 kcal/mol.

In a study, Snoussi et al. (2021) used *Allium subhirsutum* L. extracts to inhibit furin protein and found that the top peptides against furin protein were Thr–Leu–Trp (−9.6 kcal/mol) and Gln–Phe–Tyr (−9.4 kcal/mol). Peptide Thr–Leu–Trp showed van der Waals (Gly265, Pro266, Gly307, Asn310, Tyr313, Asp530, Ala532, Tyr571), attractive charge/salt bridge/Pi-anion (Glu271, Trp531), conventional hydrogen bond (Ser311, Gln48), and unfavorable positive–positive/donor–donor (Ile312, Arg490) interactions. The second peptide Gln–Phe–Tyr exhibited van der Waals (Asp154, His194, Val231, Trp254, Asp258, Asp264, Gly265, Pro266, Gly294), conventional hydrogen bond (Ser253, Gly255, Asn295, Tyr308, Ser368), Pi-anion (Glu236), and Pi-alkyl (Leu227, Pro256) (Snoussi et al., 2021). On the other hand, Asn310 and Ala532 showed H-bonding in our top compounds, robustaflavone and amentoflavone complexes, and these residues also showed van der Waal interaction in Thr–Leu–Trp peptide complex with furin. Whereas, Glu271 also showed H-bond formation in both robustaflavone and amentoflavone but was involved in attractive charge/salt bridge/Pi-anion interaction of only Thr–Leu–Trp peptide complex. Additionally, Asp264 and Glu265 residues were both involved in the H-bonding of robustaflavone, but Glu265 showed van der Waals interaction in both the peptides Thr–Leu–Trp and Gln–Phe–Tyr, whereas Asp264 showed van der Waals interaction only in Gln–Phe–Tyr peptide complex.

## Conclusion

Through high-throughput virtual screening, a library of 408 phytochemical compounds underwent rigorous screening to identify potential inhibitors of human furin protein. This process led to the finding of the top three compounds: withanolide, robustaflavone, and amentoflavone. Furthermore, the analysis included simulating the dynamic conformations of furin with these compounds. Over a period of 100 ns, these simulations revealed the formation of a persistent stable complex between furin and the identified compounds due to their structural diversity and strong binding affinity. Hence, these three phytochemicals may be potential candidates for targeted therapy in SARS-CoV-2 patients. However, their efficacy and safety should be validated through *in vivo* and *in vitro* studies before being considered for clinical use.

## Data availability statement

The original contributions presented in the study are included in the article/**Supplementary Material**. Further inquiries can be directed to the corresponding authors.

## Author contributions

PK: Writing – original draft, Validation, Software, Methodology, Writing – review & editing, Data curation. MC: Writing – review & editing, Validation, Data curation. RM: Writing – review & editing. SG: Writing – review & editing. AC: Writing – review & editing. MA-Z: Writing – review & editing. AA: Writing – review & editing. FN: Writing – review & editing. NJ: Writing – review & editing. KP: Writing – review & editing. MK: Writing – review & editing. SK: Supervision, Data curation, Conceptualization, Writing – review & editing.
