# Supplementary material for: Structure-based virtual screening methods for the identification of novel phytochemical inhibitors targeting furin protease for the management of COVID-19
Source: Front Cell Infect Microbiol. 2024 Jun 11;14:1391288. doi: 10.3389/fcimb.2024.1391288 (PMC11196402; doi:10.3389/fcimb.2024.1391288)
Supplement: Supplementary file 1 [file DataSheet_1.docx]

**
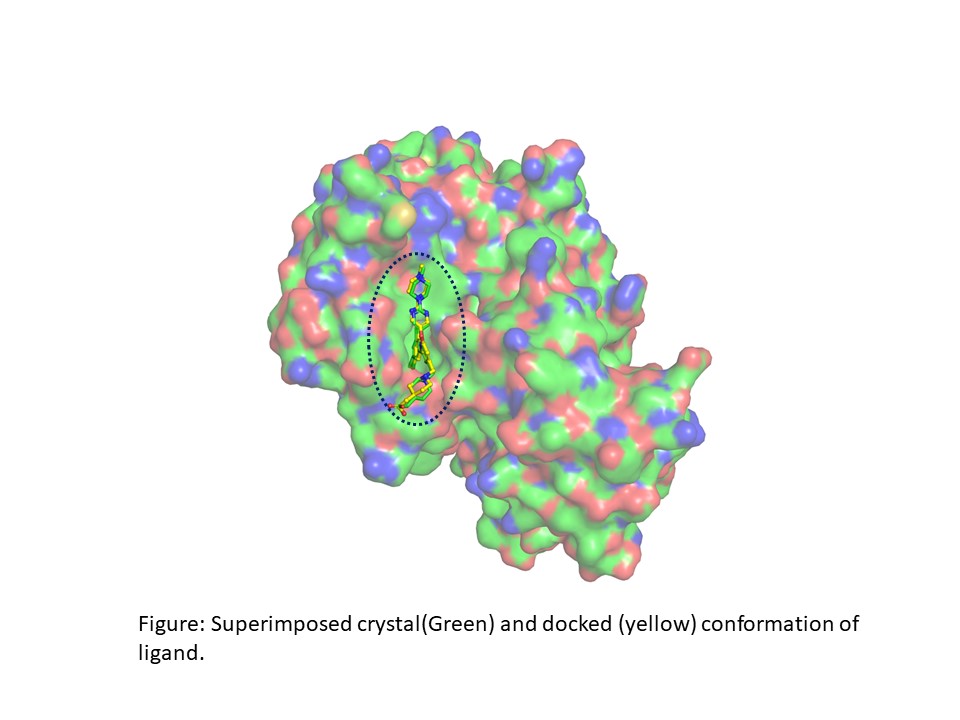
Supplementary Figure S1.**

**Supplementary Figure S1.** Surface view of furin protease depicts binding pocket (circled). Docked reference molecule pose (yellow stick) superimposed with crystal complex conformation (green stick), detailed in the manuscript

**Table S1.** List of virtual screened phytochemical compounds with antiviral property against Furin (5 MIM) protein of Human

| Ligand | Binding Affinity |
| --- | --- |
| 5281694 | -10.7 |
| 5281600 | -10.5 |
| 53477765 | -10.4 |
| 3663 | -10.1 |
| 15137997 | -9.9 |
| 5271805 | -9.8 |
| 64945 | -9.8 |
| 129905285 | -9.7 |
| 471426 | -9.7 |
| 10206 | -9.6 |
| 115250 | -9.6 |
| 6419835 | -9.5 |
| 73145 | -9.5 |
| 265237 | -9.5 |
| 5488801 | -9.4 |
| 129905286 | -9.4 |
| 73568 | -9.4 |
| 65064 | -9.4 |
| 73481 | -9.4 |
| 131676073 | -9.4 |
| 73659 | -9.4 |
| 135403798 | -9.4 |
| 101520 | -9.4 |
| 10114 | -9.4 |
| 10621 | -9.3 |
| 5321162 | -9.3 |
| 54676038 | -9.2 |
| 442428 | -9.2 |
| 12313665 | -9.2 |
| 5282150 | -9.2 |
| 6857439 | -9.2 |
| 441071 | -9.2 |
| 593033 | -9.1 |
| 122724 | -9.1 |
| 162517550 | -9.1 |
| 73078 | -9.1 |
| 5281366 | -9.1 |
| 124025 | -9.1 |
| 73193 | -9.1 |
| 101502236 | -9 |
| 10480940 | -9 |
| 5281599 | -9 |
| 11892267 | -9 |
| 5281613 | -9 |
| 46881919 | -9 |
| 11059920 | -9 |
| 6443484 | -9 |
| 130976 | -9 |
| 122738 | -9 |
| 11250133 | -9 |
| 441764 | -9 |
| 92097 | -9 |
| 10411574 | -9 |
| 99474 | -8.9 |
| 57391949 | -8.9 |
| 10177 | -8.9 |
| 439501 | -8.9 |
| 31553 | -8.9 |
| 11982272 | -8.9 |
| 6474310 | -8.8 |
| 5281612 | -8.8 |
| 5281855 | -8.8 |
| 185465 | -8.8 |
| 91472 | -8.8 |
| 5889042 | -8.8 |
| 5280445 | -8.8 |
| 9913968 | -8.8 |
| 56843093 | -8.8 |
| 466314 | -8.8 |
| 5281867 | -8.8 |
| 5320532 | -8.7 |
| 5281605 | -8.7 |
| 64971 | -8.7 |
| 5317051 | -8.7 |
| 5281773 | -8.7 |
| 15735849 | -8.7 |
| 14849116 | -8.7 |
| 442439 | -8.7 |
| 10343070 | -8.7 |
| 159516 | -8.7 |
| 5281697 | -8.7 |
| 11787114 | -8.7 |
| 5213 | -8.7 |
| 131676072 | -8.7 |
| 38347030 | -8.7 |
| 24360 | -8.7 |
| 182232 | -8.6 |
| 5280704 | -8.6 |
| 64982 | -8.6 |
| 5281607 | -8.6 |
| 21672638 | -8.6 |
| 5481663 | -8.6 |
| 168849 | -8.6 |
| 11729855 | -8.6 |
| 5280794 | -8.6 |
| 5280443 | -8.5 |
| 101526067 | -8.5 |
| 6449879 | -8.5 |
| 5316097 | -8.5 |
| 5281406 | -8.5 |
| 5280637 | -8.5 |
| 6712545 | -8.5 |
| 9548717 | -8.5 |
| 5281718 | -8.5 |
| 107876 | -8.5 |
| 65380 | -8.5 |
| 5280805 | -8.5 |
| 131975 | -8.5 |
| 131750183 | -8.5 |
| 5280442 | -8.4 |
| 5280746 | -8.4 |
| 222284 | -8.4 |
| 15944778 | -8.4 |
| 184311 | -8.4 |
| 44457257 | -8.4 |
| 72376 | -8.4 |
| 108065 | -8.4 |
| 5281806 | -8.4 |
| 5462447 | -8.4 |
| 185617 | -8.4 |
| 10207 | -8.3 |
| 441647 | -8.3 |
| 10208 | -8.3 |
| 128861 | -8.3 |
| 10429214 | -8.3 |
| 3220 | -8.3 |
| 40580807 | -8.3 |
| 73065 | -8.3 |
| 5281673 | -8.3 |
| 441773 | -8.3 |
| 443740 | -8.3 |
| 6602508 | -8.3 |
| 114917 | -8.3 |
| 164676 | -8.3 |
| 71659628 | -8.3 |
| 9851101 | -8.3 |
| 10569 | -8.2 |
| 162305 | -8.2 |
| 160254 | -8.2 |
| 107971 | -8.2 |
| 119454 | -8.2 |
| 54711004 | -8.2 |
| 11425923 | -8.2 |
| 5281368 | -8.2 |
| 3503 | -8.2 |
| 72281 | -8.2 |
| 513197 | -8.2 |
| 259846 | -8.2 |
| 5318767 | -8.2 |
| 5320686 | -8.2 |
| 185904 | -8.1 |
| 2353 | -8.1 |
| 9064 | -8.1 |
| 969516 | -8.1 |
| 124052 | -8.1 |
| 73062 | -8.1 |
| 443013 | -8.1 |
| 72378 | -8.1 |
| 442813 | -8.1 |
| 68071 | -8.1 |
| 5280459 | -8.1 |
| 5281792 | -8.1 |
| 101999460 | -8.1 |
| 73549 | -8.1 |
| 10022766 | -8.1 |
| 440832 | -8.1 |
| 8550 | -8.1 |
| 6474640 | -8 |
| 5317644 | -8 |
| 1550607 | -8 |
| 12305761 | -8 |
| 15559239 | -8 |
| 5281377 | -8 |
| 187808 | -8 |
| 637394 | -8 |
| 122216365 | -8 |
| 5281650 | -8 |
| 5281672 | -8 |
| 932 | -8 |
| 7251185 | -8 |
| 6857438 | -8 |
| 94162 | -8 |
| 71659627 | -8 |
| 5316673 | -8 |
| 72276 | -7.9 |
| 5318517 | -7.9 |
| 361042 | -7.9 |
| 22169421 | -7.9 |
| 64972 | -7.9 |
| 145714515 | -7.9 |
| 5469424 | -7.9 |
| 5317750 | -7.9 |
| 5281643 | -7.9 |
| 10215 | -7.9 |
| 3084066 | -7.9 |
| 638024 | -7.9 |
| 11220670 | -7.9 |
| 189065 | -7.8 |
| 1779468 | -7.8 |
| 442027 | -7.8 |
| 457801 | -7.8 |
| 503731 | -7.8 |
| 156582602 | -7.8 |
| 5281807 | -7.8 |
| 72946627 | -7.8 |
| 46173922 | -7.8 |
| 102335850 | -7.8 |
| 71659767 | -7.8 |
| 65188 | -7.8 |
| 10399864 | -7.8 |
| 536302 | -7.7 |
| 270972 | -7.7 |
| 20836500 | -7.7 |
| 155094 | -7.7 |
| 5282102 | -7.7 |
| 5490064 | -7.7 |
| 10337211 | -7.7 |
| 53323205 | -7.7 |
| 6441419 | -7.7 |
| 145937 | -7.7 |
| 5281614 | -7.7 |
| 7082474 | -7.7 |
| 5281255 | -7.7 |
| 129316816 | -7.7 |
| 667639 | -7.7 |
| 5321987 | -7.7 |
| 72326 | -7.6 |
| 101250074 | -7.6 |
| 44590821 | -7.6 |
| 124021 | -7.6 |
| 5281665 | -7.6 |
| 3884 | -7.6 |
| 5481964 | -7.6 |
| 7075765 | -7.6 |
| 5280537 | -7.6 |
| 5281678 | -7.6 |
| 5281680 | -7.6 |
| 5280804 | -7.6 |
| 5280343 | -7.6 |
| 16655065 | -7.6 |
| 131753040 | -7.6 |
| 10022050 | -7.6 |
| 676299 | -7.5 |
| 160190 | -7.5 |
| 1794427 | -7.5 |
| 5372945 | -7.5 |
| 5281708 | -7.5 |
| 656516 | -7.5 |
| 398941 | -7.5 |
| 5281400 | -7.5 |
| 5280961 | -7.5 |
| 276389 | -7.5 |
| 453214 | -7.5 |
| 445154 | -7.5 |
| 6481824 | -7.5 |
| 439533 | -7.5 |
| 73353398 | -7.5 |
| 5280633 | -7.4 |
| 636848 | -7.4 |
| 10366055 | -7.4 |
| 174174 | -7.4 |
| 66065 | -7.4 |
| 92765 | -7.4 |
| 6602378 | -7.4 |
| 5318717 | -7.4 |
| 5281670 | -7.4 |
| 10146 | -7.4 |
| 10914547 | -7.4 |
| 10607 | -7.4 |
| 49771359 | -7.4 |
| 68229 | -7.4 |
| 68077 | -7.4 |
| 71659765 | -7.4 |
| 11848155 | -7.3 |
| 444795 | -7.3 |
| 307918 | -7.3 |
| 480860 | -7.3 |
| 5280544 | -7.3 |
| 73467 | -7.3 |
| 160472 | -7.3 |
| 72344 | -7.3 |
| 10411189 | -7.3 |
| 92114 | -7.3 |
| 5281703 | -7.3 |
| 431012 | -7.3 |
| 71659766 | -7.3 |
| 10133609 | -7.2 |
| 88881 | -7.2 |
| 261859 | -7.2 |
| 65366 | -7.2 |
| 641785 | -7.2 |
| 551469 | -7.2 |
| 154417 | -7.2 |
| 441564 | -7.2 |
| 6610292 | -7.2 |
| 193296 | -7.2 |
| 290897 | -7.1 |
| 99856 | -7.1 |
| 128913 | -7.1 |
| 656498 | -7.1 |
| 389002 | -7.1 |
| 108058 | -7.1 |
| 5281544 | -7.1 |
| 442432 | -7.1 |
| 4680 | -7.1 |
| 11380920 | -7.1 |
| 66548 | -7.1 |
| 85152699 | -7.1 |
| 150893 | -7 |
| 124319 | -7 |
| 644019 | -7 |
| 135727631 | -7 |
| 5281616 | -7 |
| 107848 | -7 |
| 9601115 | -7 |
| 5280863 | -7 |
| 6440659 | -7 |
| 91434 | -7 |
| 10582671 | -7 |
| 5468522 | -6.9 |
| 1548943 | -6.9 |
| 6253344 | -6.9 |
| 101976765 | -6.9 |
| 261166 | -6.9 |
| 6440400 | -6.9 |
| 101324862 | -6.9 |
| 6911854 | -6.9 |
| 633619 | -6.8 |
| 9651 | -6.8 |
| 5485207 | -6.8 |
| 72303 | -6.8 |
| 6430534 | -6.7 |
| 176911 | -6.7 |
| 68486 | -6.7 |
| 54445 | -6.6 |
| 3218 | -6.6 |
| 7428 | -6.6 |
| 10189 | -6.5 |
| 6199 | -6.5 |
| 179620 | -6.5 |
| 621354 | -6.5 |
| 439503 | -6.5 |
| 101998817 | -6.4 |
| 70697809 | -6.4 |
| 370 | -6.4 |
| 5353609 | -6.3 |
| 689043 | -6.2 |
| 445858 | -6.2 |
| 442134 | -6.2 |
| 5281794 | -6.2 |
| 51683 | -6.2 |
| 637540 | -6.1 |
| 7424 | -6.1 |
| 11230 | -6.1 |
| 64961 | -6.1 |
| 10364 | -6.1 |
| 287064 | -6.1 |
| 95779 | -6.1 |
| 72 | -6 |
| 637541 | -6 |
| 54670067 | -6 |
| 323 | -6 |
| 444539 | -5.9 |
| 3652 | -5.9 |
| 381152 | -5.9 |
| 8468 | -5.9 |
| 3016110 | -5.9 |
| 3469 | -5.8 |
| 8768 | -5.8 |
| 5282826 | -5.8 |
| 442793 | -5.8 |
| 54675850 | -5.8 |
| 338 | -5.8 |
| 10281 | -5.8 |
| 5366244 | -5.7 |
| 579163 | -5.7 |
| 2758 | -5.6 |
| 1254 | -5.6 |
| 7463 | -5.6 |
| 637775 | -5.6 |
| 2519 | -5.5 |
| 638011 | -5.5 |
| 126 | -5.4 |
| 2719 | -5.4 |
| 5280435 | -5.4 |
| 22311 | -5.3 |
| 5280450 | -5.3 |
| 8655 | -5.3 |
| 10742 | -5.3 |
| 875 | -5.3 |
| 6549 | -5.2 |
| 445639 | -5.2 |
| 26136 | -4.8 |
| 10268 | -4.7 |
| 4650 | -4.6 |
| 5386591 | -4.6 |
| 65036 | -4.3 |
| 5350 | -4.3 |
| 68152 | -4 |
| 129712276 | -4 |
| 8857 | -3.8 |
| 1118 | -3.7 |
| 16590 | -3.6 |
| 11617 | -3.6 |
| 16315 | -3.6 |
| 8030 | -3 |
| 6212 | -2.6 |
| 13367 | -2.5 |

**Table S2.** ADME analysis for the top four selected phytochemical compounds as inhibitor against furin protein

| **PROPERTIES** | **Robustaflavone** | **Amentoflavone** | **Withanolide** |
| --- | --- | --- | --- |
| Molecular weight | 538.46 | 538.46 | 470.6 |
| Heavy atoms | 40 | 40 | 34 |
| Aromatic heavy atoms | 32 | 32 | 0 |
| Fraction Csp3 | 0 | 0 | 0.79 |
| Rotatable bonds | 3 | 3 | 2 |
| H-bond acceptors | 10 | 10 | 6 |
| H-bond donors | 6 | 6 | 2 |
| MR | 146.97 | 146.97 | 127.53 |
| TPSA | 181.8 | 181.8 | 96.36 |
| iLOGP | 3.06 | 3.06 | 3.78 |
| XLOGP3 | 5.04 | 5.04 | 3.12 |
| WLOGP | 5.13 | 5.13 | 3.5 |
| MLOGP | 0.25 | 0.25 | 2.75 |
| Silicos-IT Log P | 4.61 | 4.61 | 3.78 |
| Consensus Log P | 3.62 | 3.62 | 3.39 |
| ESOL Log S | -6.75 | -6.75 | -4.59 |
| ESOL Solubility (mg/ml) | 9.63E-05 | 9.63E-05 | 1.21E-02 |
| ESOL Solubility (mol/l) | 1.79E-07 | 1.79E-07 | 2.56E-05 |
| ESOL Class | Poorly soluble | Poorly soluble | Moderately soluble |
| Ali Log S | -8.6 | -8.6 | -4.81 |
| Ali Solubility (mg/ml) | 1.36E-06 | 1.36E-06 | 7.25E-03 |
| Ali Solubility (mol/l) | 2.52E-09 | 2.52E-09 | 1.54E-05 |
| Ali Class | Poorly soluble | Poorly soluble | Moderately soluble |
| Silicos-IT LogSw | -8.7 | -8.7 | -3.78 |
| Silicos-IT Solubility (mg/ml) | 1.07E-06 | 1.07E-06 | 7.85E-02 |
| Silicos-IT Solubility (mol/l) | 1.98E-09 | 1.98E-09 | 1.67E-04 |
| Silicos-IT class | Poorly soluble | Poorly soluble | Soluble |
| GI absorption | Low | Low | High |
| BBB permeant | No | No | No |
| Pgp substrate | No | No | Yes |
| CYP1A2 inhibitor | No | No | No |
| CYP2C19 inhibitor | No | No | No |
| CYP2C9 inhibitor | No | No | No |
| CYP2D6 inhibitor | No | No | No |
| CYP3A4 inhibitor | No | No | No |
| log Kp (cm/s) | -6.01 | -6.01 | -6.96 |
| Lipinski violations | 2 | 2 | 0 |
| Ghose violations | 2 | 2 | 1 |
| Veber violations | 1 | 1 | 0 |
| Egan violations | 1 | 1 | 0 |
| Muegge violations | 3 | 3 | 0 |
| Bioavailability Score | 0.17 | 0.17 | 0.55 |
| PAINS alerts | 0 | 0 | 0 |
| Brenk alerts | 0 | 0 | 1 |
